# Improving Trauma Data Quality in Rwanda: A Prospective Assessment of Registry Completeness and Accuracy

**DOI:** 10.3390/jcm15145640

**Published:** 2026-07-18

**Authors:** Jean Nepomuscene Ntezimana, Indrakantha Welgama, Assuman Nuhu, Irene Bagahirwa, Venantie Umuhoza, Fabrice Iradukunda, Leila Ghalichi, Ephrem Daniel Sheferaw, Justine Ina Davies, Jean Claude Byiringiro

**Affiliations:** 1 College of Medicine and Health Sciences, University of Rwanda, Kigali P.O. Box 4285, Rwanda; nuhu.assuman@ur.ac.rw (A.N.); j.byiringiro@ur.ac.rw (J.C.B.); 2 Department of Applied Health Sciences, University of Birmingham, Birmingham B15 2TT, UK; i.p.welgama@bham.ac.uk (I.W.); leila.ghalichi@bham.ac.uk (L.G.); j.davies.6@bham.ac.uk (J.I.D.); 3 Rwanda Biomedical Centre, Ministry of Health, Kigali P.O. Box 7162, Rwanda; irene.bagahirwa2013@gmail.com (I.B.); venantieumuhoza2@gmail.com (V.U.); fabrice.iradukunda@rbc.gov.rw (F.I.); 4 Center for Equity in Global Surgery, University of Global Health Equity, Kigali P.O. Box 6955, Rwanda; 5 Centre for Global Surgery, Stellenbosch University, Stellenbosch 7600, South Africa

**Keywords:** data quality, case missingness, variable missingness, variable accuracy, trauma registry

## Abstract

**Background**: High-quality trauma data are essential for data-informed decision-making. However, completeness and accuracy of trauma registries remain challenging in low- and middle-income countries. This study aimed to assess trauma registry data quality in Rwanda and describe a coordinated implementation package used to support improvements in registry completeness and accuracy. **Methods**: We conducted a prospective, longitudinal quasi-experimental quality-improvement study with repeated monthly measurements across eight hospitals in Rwanda from October 2023 to September 2025. Trauma registry data quality indicators—case missingness, variable missingness, and variable accuracy—were assessed monthly using a predefined Rwanda Trauma Registry data quality assessment and reporting protocol. **Results**: A total of 26,268 trauma cases were recorded across the eight hospitals. Linear mixed-effects models showed significant temporal improvements in trauma registry data quality. Case missingness decreased by 0.72 percentage points per month (β = −0.721; 95% CI −0.955 to −0.487; *p* < 0.001), and variable missingness declined by 0.33 percentage points per month (β = −0.331; 95% CI −0.435 to −0.228; *p* < 0.001). Variable accuracy increased by 0.25 percentage points per month (β = 0.253; 95% CI 0.198 to 0.307; *p* < 0.001). **Conclusions**: The findings show substantial improvements in registry completeness and accuracy during a coordinated, bundled, PDSA-guided quality-improvement implementation process in Rwanda. Because the study was uncontrolled and the activities were implemented together, the results should be interpreted as associations over time rather than causal effects of any individual intervention component.

## 1. Background

Trauma is a significant global public health concern, responsible for nearly 4.8 million deaths each year, which account for 10% of global mortality, surpassing fatalities from diseases such as tuberculosis, malaria, and HIV/AIDS combined [1]. The burden of injury is disproportionately borne by low- and middle-income countries (LMICs), where more than 90% of global trauma-related deaths occur [2]. In Rwanda, the burden of injury remains substantial, contributing to an estimated 9% of all deaths and 10% of disability-adjusted life years (DALYs) nationwide [3]. The World Health Organization (WHO) emphasizes that effective injury prevention and improved trauma care depend on evidence-based strategies tailored to each country’s context. Achieving this requires comprehensive and reliable data capturing all key aspects of trauma patient care [4].

Effective trauma care systems rely on high-quality data for surveillance, policy development, and continuous quality improvement. Trauma registries have proven indispensable in this context, providing structured, patient-level data that inform clinical governance, benchmarking, and injury prevention strategies [5]. However, the adoption and sustainability of such registries in LMICs face considerable challenges, including limited funding and human resources, fragmented administrative support, and insufficient infrastructure [6]. These challenges can lead to poor data quality, which limits the usefulness of the data that is collected.

Despite the recognized value of trauma registries, few LMICs have established nationally standardized systems [2]. Among the 115 countries reporting health data to the World Health Organization (WHO), only 29 had comprehensive national trauma registries; in many LMICs, registry development remains a low priority in the absence of policy integration and sustainable funding mechanisms [2].

In Rwanda, the WHO Trauma Registry (TR) was introduced in 2019 across selected tertiary and secondary hospitals under the coordination of the Rwanda Biomedical Centre (RBC). The TR was initially supported by funding from the United States National Institutes of Health (NIH) between September 2019 and September 2020 [7]. Thereafter, support resumed in March 2021 through the African Federation for Emergency Medicine (AFEM), which sustained the registry until August 2022 [7]. In October 2023, the registry was restarted with new funding from the UK National Institute for Health and Care Research (NIHR), under which it continues to operate to date. These shifts in external funding illustrate the vulnerability of registry sustainability in LMIC settings and highlight the importance of developing resilient, locally supported mechanisms for ongoing data collection and quality monitoring.

While Rwanda’s nationwide deployment of the WHO Trauma Registry marks an important milestone in injury surveillance, early studies already point to significant challenges in data completeness and accuracy that may limit the registry’s utility unless addressed. For example, in a study of burn unit logbooks at the University Teaching Hospital Kigali, 64.2% of records had incomplete data between 2005 and 2019; commonly missing fields included referral status and surgical treatment [8]. Similarly, in the Epidemiology of Pediatric Injuries study based in Rwanda, periodic data audits identified missingness and variable inaccuracies; however, these reviews were intermittent and relied on laborious chart reviews [9]. These findings suggest that training, funding, and systematic processes (e.g., regular audits, feedback loops) are likely to be major determinants of improving data quality over time in Rwanda [7].

The goal of strengthening trauma data systems in Rwanda is to generate locally relevant evidence that can guide registry implementation and ensure that the trauma registry reliably supports clinical decision-making, health system planning, and national injury prevention efforts. The specific purpose of this study was to assess trauma registry completeness and accuracy over time and to describe the practical implementation processes used to identify data-quality gaps and support improvement across hospitals. The contribution of this paper is therefore not only the reporting of data-quality trends, but also the description of a nationally coordinated, protocol-based approach to trauma registry monitoring in an LMIC setting, including how hospital registrars, a research team, and the Rwanda Biomedical Centre worked together to review registry performance, identify implementation barriers, and recommend corrective actions. Building on the documented challenges of missing and inaccurate trauma data in Rwanda, this study provides implementation evidence that may inform countries seeking to strengthen trauma surveillance systems under similar resource constraints.

## 2. Methodology

### 2.1. Study Design

We conducted a prospective, multicenter, quasi-experimental, longitudinal quality-improvement study with repeated monthly measurements to assess trauma registry data quality over time. The study was embedded within routine trauma registry implementation and quality-improvement activities and did not include randomization or a parallel control group. The intervention was implemented as a bundled and adaptive package rather than as separate, sequentially tested components; therefore, the study was not designed to establish causality or to estimate the independent effect of any single component. Findings are interpreted as temporal changes in data quality indicators observed during implementation.

### 2.2. Study Setting

The study was conducted across eight hospitals implementing the WHO Clinical Registry (Trauma Module) in Rwanda, including one district hospital, one provincial hospital, three level II teaching hospitals, and three tertiary referral hospitals with emergency departments. These hospitals represent diverse levels of care and geographic settings. The study period spanned October 2023 to September 2025, allowing for assessment of data quality trends over an extended implementation period. Table 1 presents the characteristics of the hospitals included in Rwanda’s trauma registry implementation, including facility name, level of care, geographic location, and the date of registry commencement. This diversity of facility types and geographic locations provides important context for interpreting variations in trauma registry data quality and emergency care processes across different hospital settings in Rwanda.

### 2.3. Trauma Registry Description

The World Health Organization Clinical Registry—Trauma Module is a standardized, web-based platform designed to support hospitals in collecting and analyzing data on injured patients. It captures uniform information across the continuum of care, including patient demographics, injury characteristics, emergency management, and outcomes, as part of national efforts to strengthen injury surveillance and improve the quality of trauma care [10].

Data are inputted into the registry by trauma registrars who were nurses employed clinically in the Emergency Departments in each hospital and trained on the structure and objectives of the WHO Trauma Module, procedures for identifying eligible trauma patients in the emergency department (ED), and standardized data entry into the electronic registry. Each hospital had two to three registrars.

At the hospital level, trauma cases were prospectively identified from ED admission registers and patient records. Relevant information was then abstracted from source documents and entered into the WHO Clinical Registry—Trauma Module using the standardized electronic platform.

### 2.4. Trauma Registry Data Quality Domains and Their Definitions

Trauma registry data quality was assessed using three metrics: case missingness, variable missingness, and variable accuracy. These metrics were selected by the authors as those identified previously with poor performance and noted to be essential for data usability; they are based on valid models for assessing data quality [11]. Case missingness was defined as the proportion of eligible trauma cases presenting to the emergency department that were not captured in the trauma registry. Variable missingness was defined as the proportion of required registry variables that were missing among recorded cases. Variable accuracy was defined as the agreement between trauma registry entries and source documents, including emergency department registers and patient clinical records. See below for analysis methods.

### 2.5. Data Collection Procedures and Eligibility Criteria

Case missingness and variable missingness were derived using data collected into the WHO Clinical Registry—Trauma Module in Rwanda. For this study, anonymized trauma registry data were extracted monthly from the registry platform for each participating hospital. Routine registry data entry was performed at the hospital level by trained trauma registrars assigned to record variables for eligible ED trauma patients into the Trauma Registry. An eligible trauma case was defined as a trauma patient presenting to the Emergency Room/Emergency Department either directly or via the outpatient department for emergency assessment or management. Trauma-related admissions from outpatient departments, patients managed only as outpatients, and patients admitted directly to other admission units outside the ED were not considered eligible for Trauma Registry entry. Duplicate records and follow-up visits for the same injury episode were excluded. Transfers into the ED were included when they represented an eligible ED trauma presentation during the reporting month, and deaths on arrival were included if they were recorded as trauma presentations in the ED register. ED registers were used as the operational reference source for case ascertainment because they are the routine hospital-level record of ED trauma presentations.

During site visits, assessment of trauma data quality status, identification of quality-related issues, and formulation of recommendations were conducted by the research team together with the Rwanda Biomedical Centre (RBC) team. RBC is a branch of the Rwanda Ministry of Health and the national central health implementation agency responsible for designing and executing public health programs, preventing diseases, and improving healthcare delivery across Rwanda. The involvement of RBC provided national oversight and supported standardized assessment and reporting across hospitals. The assessment team reviewed ED registers to identify eligible trauma presentations for comparison with registry entries and, when possible, checked register counts against available triage logs, admission books, and patient files to identify obvious duplication or miscoding. For variable accuracy, sampled registry records were cross-validated against ED admission registers and patient source documents (paper records or electronic health records).

### 2.6. Quality Improvement Interventions

To support continuous improvement in trauma registry data quality, the study team implemented an iterative quality-improvement approach guided by the Plan–Do–Study–Act (PDSA) framework. Site visits were conducted monthly or every two months, depending on hospital performance, with more frequent visits maintained for hospitals with persistent data quality gaps. Visits were conducted by the research team in collaboration with the Rwanda Biomedical Centre (RBC), the national health implementation agency under the Rwanda Ministry of Health.

During site visits, the research and RBC teams assessed trauma registry data quality status, identified barriers to complete and accurate documentation, and developed site-specific recommendations. Findings were then shared with hospital stakeholders, including trauma registrars, ED physicians, nurses, matrons, and the quality improvement focal person. Feedback meetings were used to discuss missed cases, incomplete variables, inconsistencies between clinical records and registry entries, and corrective actions.

The PDSA process was applied pragmatically: monthly data-quality reports were reviewed to identify problems, corrective actions were implemented with hospital teams, subsequent indicators were reviewed to assess progress, and successful practices were reinforced while persistent gaps informed further follow-up. Initial registrar training focused on case identification, registry variable definitions, use of the WHO Clinical Registry—Trauma Module, data-entry procedures, dashboard use, and resolving discrepancies between source documents and registry entries. Refresher training was conducted after one year to address recurring challenges and strengthen registry use for local improvement.

Implementation was monitored through scheduled site visits, monthly data-quality reports, documentation of identified challenges, and follow-up of agreed action points. Although no formal fidelity score was collected, standardized reporting procedures and repeated feedback supported consistency across hospitals while allowing adaptation to local context. Refer to Table 2, these interventions were implemented throughout the study period, with monthly monitoring of trauma registry performance indicators to evaluate changes in case capture, variable missingness, and variable accuracy over time.

The implementation of quality-improvement interventions followed a phased and adaptive approach in response to evolving data quality challenges across participating hospitals. Initial efforts focused on standardizing case identification and registry use, followed by progressive strengthening through routine data quality audits, feedback mechanisms, and stakeholder engagement. Over time, targeted supervision and operational support were introduced to address persistent gaps, while lessons from early implementation informed the scale-up to additional hospitals where the trauma registry was introduced (Table 3).

### 2.7. Data Analysis

Trauma Registry data for the study period were downloaded in anonymized form, reviewed, and cleaned to eliminate duplicate entries. Descriptive analyses were done in STATA version 18. Monthly site-specific trends were examined.

**(a) Variable Missingness**: this was assessed as the percentage of missing values across a set of 52 variables selected due to their importance to the local service providers; skip patterns were accounted for in the analysis. Variables of importance were agreed upon in a workshop of RBC, facility, and emergency department leads in 2023. For each case, **TV** = total number of expected applicable variable fields among recorded trauma cases; **CV** = number of completed applicable variable fields.

$$\mathrm{\% Variable Missingness}\;=\;(\frac{TV-CV}{TV})\;\times \;100$$

**(b) Case Missingness** was calculated as the percentage of trauma cases presenting to the ED and entered into the department registry during a given month that were not entered into the trauma registry. This was determined by comparing the number of ED trauma presentations with the number of cases captured in the registry:

$$\mathrm{\%Case Missingness}\;=\;(\frac{NH-NT}{NH})\;\times \;100$$

where **NH** = no. of eligible cases entered into the ED admission register during the month; **NT** = no. of cases entered the Trauma Registry during that same month.

**(c) Variable Accuracy** was determined by comparing registry entries with patient source documents (paper records or electronic health records) for a random monthly sample. If more than 100 cases were recorded, 10% were selected; if fewer than 100 cases were recorded, 10 were reviewed. Accuracy was expressed as the proportion of correctly recorded variables relative to the source documentation. The average percentage of the accuracy of the variable entries into the Trauma Registry was then assessed.

For each selected case,

**TV** = Total number of applicable variables checked for accuracy in sampled cases;

**AV** = Number of variables accurately entered into the Trauma Registry;

**N** = Number of selected cases (10% of all cases entered into the trauma registry for the given month).

$$\mathrm{Percentage of Accurate Entries per Case}\;=\;\frac{AV}{TV}\;\times \;100$$

$$\mathrm{Percentage accuracy}\;=\;\sum \frac{percentage\;of\;accuracy\;of\;entries\;per\;case}{N}\;\mathrm{of selected N cases}\;\;\;\;\;\;\;\;\;\;\;\;\;\;\;\;\;\;\;\;\;\;\;\;\;\;\;\;\;\;\;\;\;\;\;\;\;\;\;\;\;\;\;\;\;\;\;\;\;\;\;\;\;\;\;\;\;\;\;\;\;\;\;\;\;\;\;\;$$

Monthly data quality metrics were summarized using descriptive statistics. Linear mixed models were used to evaluate changes in data quality over time, accounting for repeated measurements and clustering at the hospital level. To assess temporal changes in data quality indicators (case missingness, variable missingness, and accuracy), we fitted linear mixed-effects models with time (in months) as a fixed effect. A random intercept for each hospital was included to account for clustering of repeated monthly observations within hospitals. Models were fitted using all available data under a missing-at-random assumption. Hospitals with incomplete data in the first year, namely Hospitals 7 and 8, were not excluded from the primary analysis; instead, their available observations from the second year were included. No additional covariates were included in the primary models, which were therefore unadjusted analyses.

As a sensitivity analysis, the linear mixed-effects models were repeated after excluding Hospitals 7 and 8, which joined the registry in October 2024 and therefore contributed only 12 months of observations. This analysis was conducted to assess whether the overall temporal trends were consistent among the six hospitals that contributed data across the full 24-month study period. Regression coefficients (β) represent the average monthly change in each data quality indicator and should not be interpreted as causal effects of individual intervention components. Results were presented with 95% confidence intervals and corresponding *p*-values. To quantify the degree of hospital-level clustering, the intraclass correlation coefficient (ICC) was estimated for each data-quality outcome from the linear mixed-effects models. The ICC represents the proportion of total variation in the outcome attributable to systematic differences between hospitals, with the remaining variation reflecting within-hospital variation across months. Higher ICC values indicate that hospital identity contributed more strongly to variation in the data-quality indicator.

## 3. Ethical Considerations

This study was conducted in accordance with the Declaration of Helsinki and relevant national and institutional guidelines. It was conducted as part of two larger studies. The first, titled “NIHR Global Health Group on Equitable Access to Quality Health Care for Injured People in Four Low- or Middle-Income Countries: Equi-injury,” received ethical approval from the Rwanda National Research Ethics Committee (ERC No. 85/RNEC/2023 and No. 14/RNEC/2024) and was approved by the Ethics Committee of the University of Birmingham. The second study, titled “Rwanda912: Use of an innovative electronic communications platform to improve pre-hospital transport of injured people in Rwanda,” received ethical approval from the Rwanda National Research Ethics Committee (No. 99/RNEC/2023). Before data collection, local institutional approvals were obtained from all participating hospitals.

**Consent to participate**: Consent to participate was waived by the Rwanda National Research Ethics Committee due to the nature of the study, which involved the use of routinely collected patient records without direct contact with participants. All data were fully anonymized before analysis to ensure confidentiality and privacy protection.

## 4. Results

**Figure 1:**
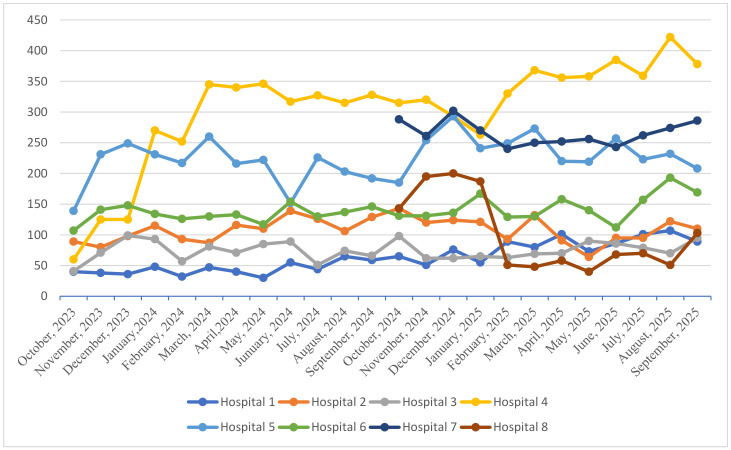
Monthly trend of trauma cases recorded in the trauma registry across participating hospitals in Rwanda (October 2023–September 2025).

During the study period, the trauma registry captured 26,268 patients across the eight participating facilities. The number of cases entered varied from 7297 cases (27.8%) in Hospital 4 to 1182 cases (4.5%) from Hospital 8 (Figure 1). Figure 1 shows the monthly trend of trauma cases recorded in the trauma registry across participating hospitals. Notably, while six hospitals reported data throughout the full study period, Hospital 7 and Hospital 8 joined the registry later, in October 2024, and thus their case volumes reflect one year of reporting.

**Figure 2:**
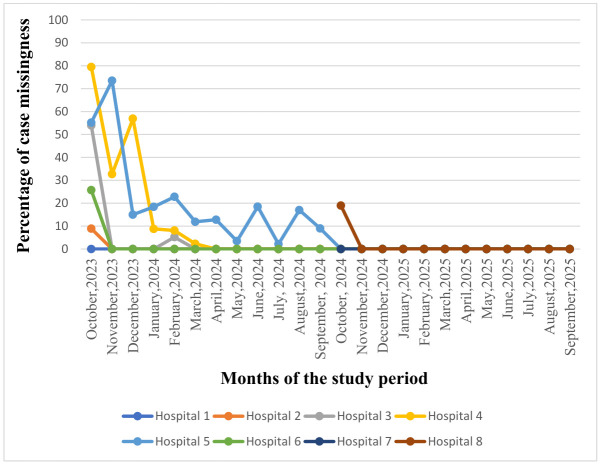
Distribution of case missingness across participating hospitals in Rwanda (October 2023–September 2025). Figure 2 presents the percentage of missing values for ED trauma cases recorded in the WHO Trauma Registry across participating facilities. Higher percentages indicate greater case missingness, reflecting lower completeness of trauma registry documentation at the respective hospital.

Case-level missingness varied substantially across hospitals and over time, with a general pattern of high missingness in the initial months of registry implementation followed by rapid improvement across all sites (Figure 2). At the beginning of data collection (October–December 2023), several hospitals exhibited extremely high missingness, with the greatest in Hospital 4 (up to 79.45%). Hospitals 1, 2, and 6 showed little to no cases of missingness during this period. By January to March 2024, missingness had declined sharply across all facilities, with most hospitals reporting 0% case missingness from mid-2024 onward. Hospital 5 showed intermittent increases during mid-2024 (e.g., 18.5% in June 2024) but subsequently stabilized at 0% like other facilities. Hospitals 7 and 8, which joined the registry later, contributed data only from late 2024 onward and demonstrated complete-case reporting (0% missingness) across all available months. From October 2024 to September 2025, all hospitals consistently achieved 0% of the missing cases.

### 4.1. Average Variable Missingness in the Trauma Registry

**Figure 3:**
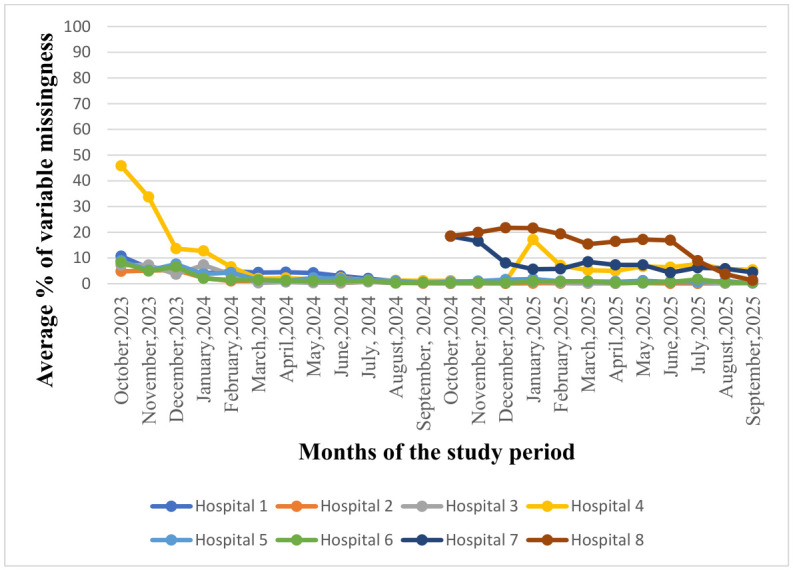
Average percentage of Variable Missingness per Hospital (October 2023–September 2025).

Across all hospitals and reporting months, the mean missingness of the variable was 4.08%, with values ranging from 0.08% to 45.81% (SD 6.34%) (Figure 3). Missingness declined steadily over time, decreasing from 14.30% in October 2023 to 1.61% by September 2025. Hospital-level performance varied, with lower average missingness in tertiary level hospitals- including Hospitals 2 (1.12%), 3 (1.56%), and 6 (1.57%), compared with district-level facilities. Moderate levels were observed in Hospitals 1 (2.38%) and 5 (2.13%), while higher rates were recorded in Hospitals 4 (8.05%), 7 (8.19%), and particularly Hospital 8 (15.08%). Hospital 4 showed the highest early variable missingness, followed by substantial improvement after intensified site supervision, targeted feedback to registrars and ED staff, and operational support.

Figure 3 shows the average percentage of missing variables for individual cases collected in the WHO Trauma Registry across participating hospitals. Higher percentages indicate greater variable missingness, reflecting reduced variable completeness and potential gaps in documentation quality within the trauma registry.

### 4.2. Accuracy of Recorded Variables

**Figure 4:**
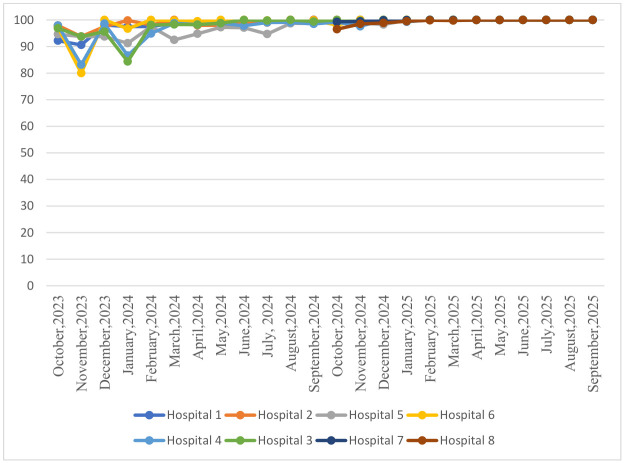
Average Percentage of Variable Accuracy Per Hospital (October 2023–September 2025).

Comparisons between trauma registry entries and corresponding patient files demonstrated consistently high variable accuracy across all participating hospitals (Table 4). Overall accuracy ranged from 80.0% to 100%, with a mean of 98.6% (SD 3.01) and a median of 99.98%. Most hospitals maintained a mean accuracy above 97%, with particularly high performance observed in Hospitals 2, 7, and 8 (≥99%). Occasional lower values were observed in earlier months in some facilities (minimum 80.0%), contributing to the wider range. As illustrated in Figure 4, accuracy improved over time, with most hospitals achieving near-complete accuracy (≥99%) in later months.

Table 5 presents the overall distribution of variable accuracy across participating hospitals. The findings indicate consistently high data accuracy across most sites, with the majority achieving near-complete performance. However, some variability exists, highlighting limited but notable inter-hospital differences.

Table 6 presents the results of the linear mixed-effects models assessing temporal changes in trauma registry data-quality indicators over the study period. The models showed statistically significant improvements in all three metrics, with reductions in case missingness and variable missingness and an increase in variable accuracy over time. These findings provide quantitative evidence of improved registry completeness and accuracy during the implementation period. However, because the study was uncontrolled and involved a bundled, adaptive quality-improvement intervention, these estimates should be interpreted as associations between time during implementation and data-quality indicators, rather than as definitive causal effects of any individual intervention component.

The intraclass correlation coefficient (ICC) was 0.000 for case missingness, 0.613 for variable missingness, and 0.000 for variable accuracy, indicating substantial hospital-level clustering for variable missingness but no meaningful detectable clustering for case missingness or variable accuracy, likely due to floor and ceiling effects.

## 5. Discussion

This prospective multicenter study demonstrates that substantial temporal improvements in trauma registry data quality can be achieved in a low-resource setting when registry implementation is accompanied by a structured and coordinated quality improvement (QI) approach. Over the 24 months, we observed marked reductions in both case and variable missingness, alongside consistently high levels of variable accuracy across participating hospitals. These findings suggest that trauma registry performance in low- and middle-income countries (LMICs) is not solely constrained by resource limitations, but is strongly influenced by governance structures, workforce capacity, and the integration of registry processes into routine clinical care [12].

Importantly, the improvements observed were not attributable to a single intervention, but rather to the cumulative effect of a phased and adaptive implementation strategy. While initial gains followed registrar training and registry reintroduction, sustained improvements were associated with ongoing supportive supervision, routine data quality audits, structured feedback, and continuous engagement with emergency department staff. This aligns with evidence from other LMIC settings, where early registry implementation is often characterized by under-reporting, followed by improvement once systematic oversight mechanisms are introduced [13,14,15]. Our findings extend this literature by demonstrating that iterative supervision and feedback are critical for maintaining and advancing data completeness over time, consistent with studies showing that audit and feedback mechanisms improve data quality in similar settings [16].

Data quality challenges were broadly similar across hospitals, though they varied in magnitude. Key barriers included inconsistent identification of eligible trauma cases, incomplete source documentation, delays in data entry, and limited familiarity with registry definitions. The observed reductions in variable missingness may reflect improvements in both registry practices and upstream clinical documentation. This is particularly relevant in LMIC contexts, where documentation is often paper-based and influenced by high clinical workloads [6]. The introduction of standardized data collection tools, a national data dictionary, and targeted training may have contributed to improved completeness, consistent with prior studies demonstrating the benefits of standardization in clinical documentation [10,14,17].

A critical consideration emerging from this study is the essential role of human and financial resources in enabling these improvements. The implementation of training programs, routine supervision, data quality audits, provision of tablets, internet connectivity support, and ongoing mentorship required sustained investment. Furthermore, the time and expertise contributed by the implementation team, including supervisors, clinicians, and data personnel, were fundamental to the success of the initiative. While strong governance and system design are key drivers of performance, these findings underscore that such structures depend on adequate resourcing. Without dedicated funding and human input, the observed improvements in registry functionality and data quality would likely not have been achievable [18,19,20]. Long-term sustainability will require progressive integration of registry activities into routine hospital and Ministry of Health systems, domestic budget lines for registry maintenance, continued training for staff turnover, and clear accountability structures after external project funding ends.

While this study was conducted in Rwanda, the implementation challenges addressed are common across many LMIC trauma registry settings, including incomplete case capture, missing variables, limited digital infrastructure, competing clinical workloads, and the need for sustained supervision and feedback [1,21]. The findings, therefore, provide practical lessons for similar health systems seeking to strengthen trauma surveillance. In particular, the study highlights the value of national coordination, standardized registrar training, routine data-quality monitoring, audit and feedback, and iterative PDSA-based follow-up. These components may be adapted by other resource-limited settings, while recognizing that the specific implementation model and level of support required will depend on local health-system structures, financing, and workforce capacity.

Finally, the sustained improvements in registry completeness, case capture, and variable accuracy demonstrate the growing maturity and practical value of Rwanda’s trauma registry. High-quality registry data are essential for credible clinical audit, performance monitoring, resource planning, injury prevention, research, and evidence-informed health policy [22,23]. The convergence of performance across hospital levels further suggests that, when supported by coordinated governance, targeted supervision, and adaptive implementation support, national trauma registries can contribute to more equitable health system strengthening [24,25,26]. The adaptive allocation of implementation support, with intensified efforts in lower-performing sites, demonstrates an efficient approach to managing resource constraints in multicenter QI initiatives [27]. These findings reinforce the importance of embedding trauma registries within well-supported national frameworks, particularly in LMICs, where reliable injury data are urgently needed to guide quality improvement and policy action [25].

### 5.1. Strength and Limitation of the Study

This prospective, quasi-experimental longitudinal quality-improvement study with repeated monthly measurements enabled systematic assessment of changes in trauma registry data quality across eight hospitals, strengthening internal validity and national relevance. Standardized definitions and routine source document verification enhanced confidence in observed improvements, while extended follow-up captured registry maturation in a low-resource setting. However, the absence of a contemporaneous control group limits causal attribution to specific intervention components. The analysis was restricted to in-hospital data, excluding pre-hospital variables. Hospitals 7 and 8 joined later than the other sites, contributing fewer observations, and residual missingness may reflect emergency department workflow constraints; findings may not generalize to settings without similar national coordination and support.

### 5.2. Research Implications

This study has important implications for trauma surveillance and health-system strengthening in LMICs. The findings suggest that trauma registry data quality can be improved through modifiable system-level strategies, including national coordination, standardized registrar training, routine data-quality assessment, structured feedback, and sustained operational support. The reduction in early performance gaps across hospitals also highlights the importance of equitable implementation support when scaling trauma registries nationally.

Reliable trauma registry data provides a foundation for clinical audit, performance monitoring, injury prevention, resource allocation, and evidence-informed trauma care policy. In practice, improved registry data can help hospitals and national teams identify missed cases, monitor documentation quality, describe injury patterns, and evaluate future trauma care interventions. Future research should examine whether improvements in registry data quality translate into better clinical processes, patient outcomes, resource use, and long-term sustainability under domestic financing mechanisms.

## 6. Conclusions

This prospective multicenter study demonstrated substantial improvements in trauma registry completeness and accuracy during a coordinated, bundled quality-improvement implementation in Rwanda. Early challenges related to case under-capture and variable missingness were progressively addressed through standardized training, supportive supervision, digital infrastructure, routine feedback, and leadership engagement. Because the study was uncontrolled and intervention components were implemented together, the findings should be interpreted as temporal associations rather than definitive causal evidence. Nevertheless, the consistently high accuracy observed across hospitals supports the potential value of trauma registries for research, clinical governance, and policy decision-making when adequate coordination, resources, and accountability mechanisms are maintained.

## Figures and Tables

**Table 1:** Description of the Hospitals where the Trauma Registry is implemented in Rwanda.

No.	Facility Level	Location	Commencement of the WHO Trauma Registry Implementation
**Hospital 1**	Level II Teaching Hospital	Eastern Province	July 2022
**Hospital 2**	Tertiary Level Care	Urban–Kigali (Kicukiro District)	August 2019
**Hospital 3**	Provincial Hospital	Western Province	June 2021
**Hospital 4**	Level II Teaching Hospital	Northern Province	August 2019
**Hospital 5**	Tertiary Level Care	Urban–Kigali (Nyarugenge District)	August 2019
**Hospital 6**	Tertiary Level Care	Southern Province	August 2019
**Hospital 7**	Level II Teaching Hospital	Urban–Kigali	October 2024
**Hospital 8**	District Hospital	Urban–Kigali	October 2024

**Table 2:** Major Problems Identified and Corresponding Interventions Implemented.

Identified Data Quality Problem	Quality Improvement Intervention	Description of Intervention	Intended Effect
Inconsistent identification of trauma cases and incorrect registry entries	Training of trauma registrars	Trauma registrars were trained on standardized case identification and accurate data entry into the WHO Clinical Registry—Trauma Module, including use of the registry platform and dashboard.	Improve correct case capture and enhance the accuracy of recorded variables.
Limited operational capacity to support real-time data entry	Provision of operational resources	Internet connectivity was facilitated, and data collection tools such as tablets and laptops were supplied to trauma registrars to support timely data entry and access to the registry dashboard.	Enable consistent data entry and improve the completeness of registry data.
Lack of awareness of data quality gaps among hospital staff	Structured feedback meetings	Findings from monthly audits were shared during structured hospital visits and feedback sessions with ED staff and trauma registrars.	Improve adherence to registry procedures and reinforce data documentation practices.
Implementation barriers and inconsistent adherence to registry procedures	Regular engagement with ED staff	Meetings with ED clinicians, matrons, and trauma registrars were conducted to discuss challenges in registry implementation and reinforce standard operating procedures.	Strengthen collaboration and improve consistency of trauma data documentation.
Limited motivation and sustainability of registry activities	Motivational support	Trauma registrars received a monthly allowance to support sustained engagement in registry data collection and entry activities.	Promote continued participation in registry activities and reduce data entry delays.
Limited institutional accountability for registry performance	Engagement of hospital leadership	Periodic presentations of site-specific findings and progress were conducted with hospital leadership to encourage oversight and local ownership of registry performance.	Strengthen accountability and institutional support for improving data quality.

**Table 3:** Phased implementation of quality-improvement interventions and observed trauma registry data quality patterns across participating hospitals, October 2023–September 2025.

Phase	Key Intervention	Hospitals Targeted	Observed Pattern
Preparation (August 2023)	Training and tools for standardized data entry	Hospitals 1–6	Established baseline procedures
Registry restart (September 2023)	Reintroduction of the WHO trauma registry	Hospitals 1–6	Initiation of prospective data collection
Early implementation (October 2023)	Audits and monthly supervision	Hospitals 1–6	High missingness followed by early decline
Team engagement (November 2023 onward)	Staff feedback meetings	Hospitals 1–6	Improved completeness and adherence
Operational strengthening (February 2024 onward)	Internet connectivity support	Hospitals 1–6	Improved timeliness and declining missingness
Adaptive supervision (April 2024 onward)	Targeted support to low-performing hospitals	Hospitals 4 and 5 received intensified supervision monthly, while Hospitals 1, 2, 3, 6 shifted to Bi-monthly	Sustained improvement and reduced gaps
Scale-up (October 2024 onward)	Early support for new hospitals	Hospitals 7 & 8	Faster improvement and high completeness
Institutionalization (October 2024 onward)	Leadership engagement	All participating hospitals	Improved ownership and sustainability

**Table 4:** Average percentage of Variable Accuracy by Hospital (October 2023–September 2025).

Hospital	Mean (%)	SD	Median (%)	Min (%)	Max (%)
**Hospital 1**	98.67	2.42	99.93	90.60	100
**Hospital 2**	99.21	1.45	100.00	93.75	100
**Hospital 3**	98.45	3.40	100.00	84.37	100
**Hospital 4**	97.81	4.18	99.08	83.20	100
**Hospital 5**	97.54	2.80	98.70	91.25	100
**Hospital 6**	98.78	4.10	100.00	80.00	100
**Hospital 7**	99.88	0.24	99.99	99.30	100
**Hospital 8**	99.41	1.04	99.93	96.50	100

**Table 5:** Overall Distribution of Variable Accuracy Across Participating Hospitals.

Statistics	Value (%)
Mean	98.6
SD	3.01
Median	99.98
Range	80–100

**Table 6:** Linear Mixed-Effects Model Estimates of Monthly trends in Trauma Registry Data Quality Indicators (October 2023–September 2025).

Outcome	Fixed Effect	β (Monthly Change)	95% CI	*p*-Value
**Case missingness (%)**	Time (per month)	−0.721	−0.955 to −0.487	<0.001
**Variable missingness (%)**	Time (per month)	−0.331	−0.435 to −0.228	<0.001
**Accuracy (%)**	Time (per month)	+0.253	+0.198 to +0.307	<0.001

***β represents the average monthly trends in each data quality indicator. Linear mixed-effects models included hospital-level random intercepts to account for clustering of repeated monthly measurements within hospitals. Negative β values indicate reductions in missingness, while positive β values indicate improvements in accuracy.***

## Data Availability

The datasets generated and/or analyzed during the current study are not publicly available due to institutional data-sharing restrictions and to protect participant confidentiality but are available from the corresponding author on reasonable request.
